# Multifocal Dedifferentiated Liposarcoma of the Jejunal Mesentery

**DOI:** 10.7759/cureus.19780

**Published:** 2021-11-21

**Authors:** Archana Khanduri, Nalini Bansal, Arvind Singh, Jyoti Gupta, Rahul Gupta

**Affiliations:** 1 Gastrointestinal Surgery, Synergy Institute of Medical Sciences, Dehradun, IND; 2 Histopathology, SRL Ltd, Fortis Escorts Heart Institute, New Delhi, IND; 3 Gastroenterology, Synergy Institute of Medical Sciences, Dehradun, IND; 4 Radiation Oncology, Swami Rama Himalayan University, Dehradun, IND

**Keywords:** jejunum, cdk 4 protein, mdm2, small bowel mesentery mass, midline laparotomy, dedifferentiated liposarcoma

## Abstract

Abdominal liposarcoma is most often located in the retroperitoneum. Small bowel mesentery is a rare site of liposarcoma. Dedifferentiated liposarcoma (DDLS) is a rare variant of mesenteric liposarcoma with only 12 cases reported in the literature. DDLS has a worse prognosis compared to a well-differentiated liposarcoma. Here, we present a case of giant liposarcoma located in the jejunal mesentery diagnosed on the contrast-enhanced computed tomography of the abdomen. The patient underwent complete surgical excision with uneventful postoperative recovery. DDLS was confirmed on histopathological examination of the resected specimen. The patient is currently receiving adjuvant therapy with doxorubicin and ifosfamide in view of close resection margin, mitotic rate and high rate of recurrence.

## Introduction

Liposarcoma is one of the commonest soft tissue sarcomas in adults. It is most often seen in the soft tissues of the extremities and retroperitoneum. It usually occurs in the fifth to seventh decades of life with a higher incidence in males. There are five main subgroups of liposarcoma: well-differentiated, myxoid, dedifferentiated, pleomorphic and myxoid pleomorphic [1]. DDLS is a rare and aggressive type of liposarcoma. Pathologically, DDLS consists of two components - atypical lipomatous tumor/well-differentiated liposarcoma and high-grade non-lipogenic sarcoma. It most commonly occurs in the retroperitoneum. Mesenteric DDLS is extremely rare with only 12 cases described in the literature [2-4]. We report a rare case of multifocal DDLS located in the jejunal mesentery treated successfully by complete surgical excision and adjuvant chemotherapy.

## Case presentation

A 55-year-old man presented with complaints of a lump in the left upper quadrant of the abdomen for two months. There was no associated fever, vomiting, abdominal pain, altered bowel habits, hematemesis or Malena. There was no significant past surgical or medical history. On clinical examination, a hard mobile lump of 9 x 9 cm was palpable in the left side of the abdomen. Routine blood investigations and tumor markers were within normal limits.

**Figure 1:**
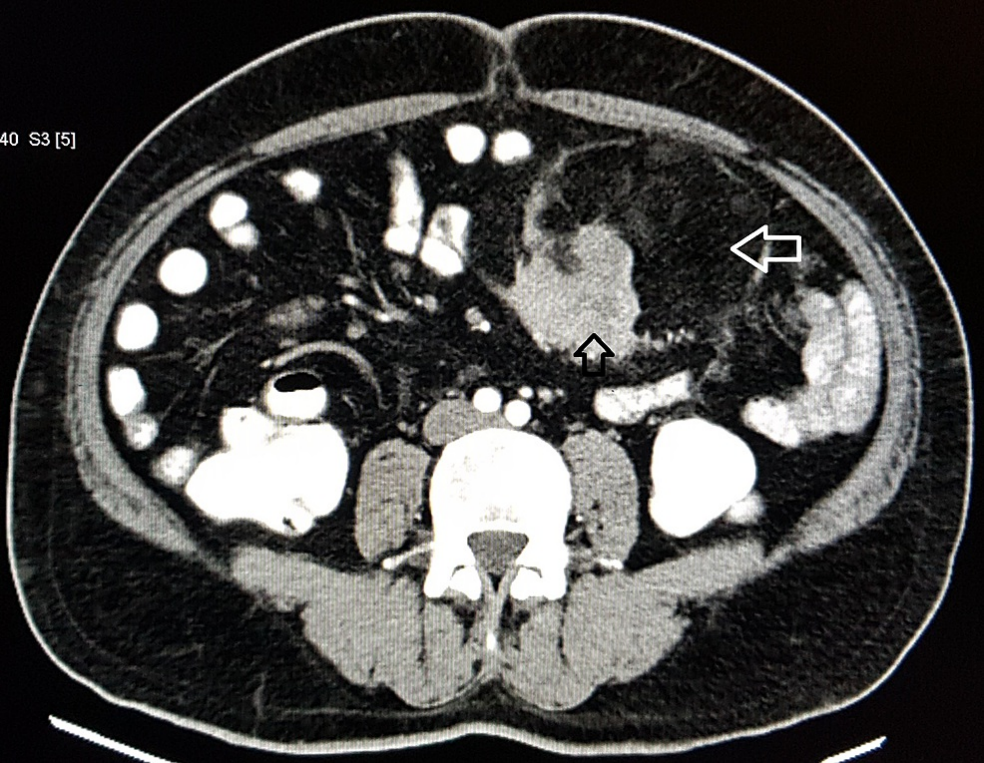
Contrast-enhanced computed tomography of the abdomen and pelvis showing the heterogenous mass with fat component (white arrow) and non-lipogenic sarcomatous component (black arrow) arising from the small bowel mesentery.

On contrast-enhanced computed tomography (CECT) of the abdomen, a large lobulated well defined complex lesion of 11 x 19 x 13 cm with heterogenous density was present in the left side of the abdomen involving the mesentery. The lesion had predominantly fat component with an enhancing soft tissue component of 5.9 x 6 x 6 cm in its inferior aspect (Figure 1). The tumor was supplied by the mesenteric vessels but there was no direct infiltration of the bowel loops by the tumor. With the provisional diagnosis of liposarcoma, the patient was planned for surgical excision.

**Figure 2:**
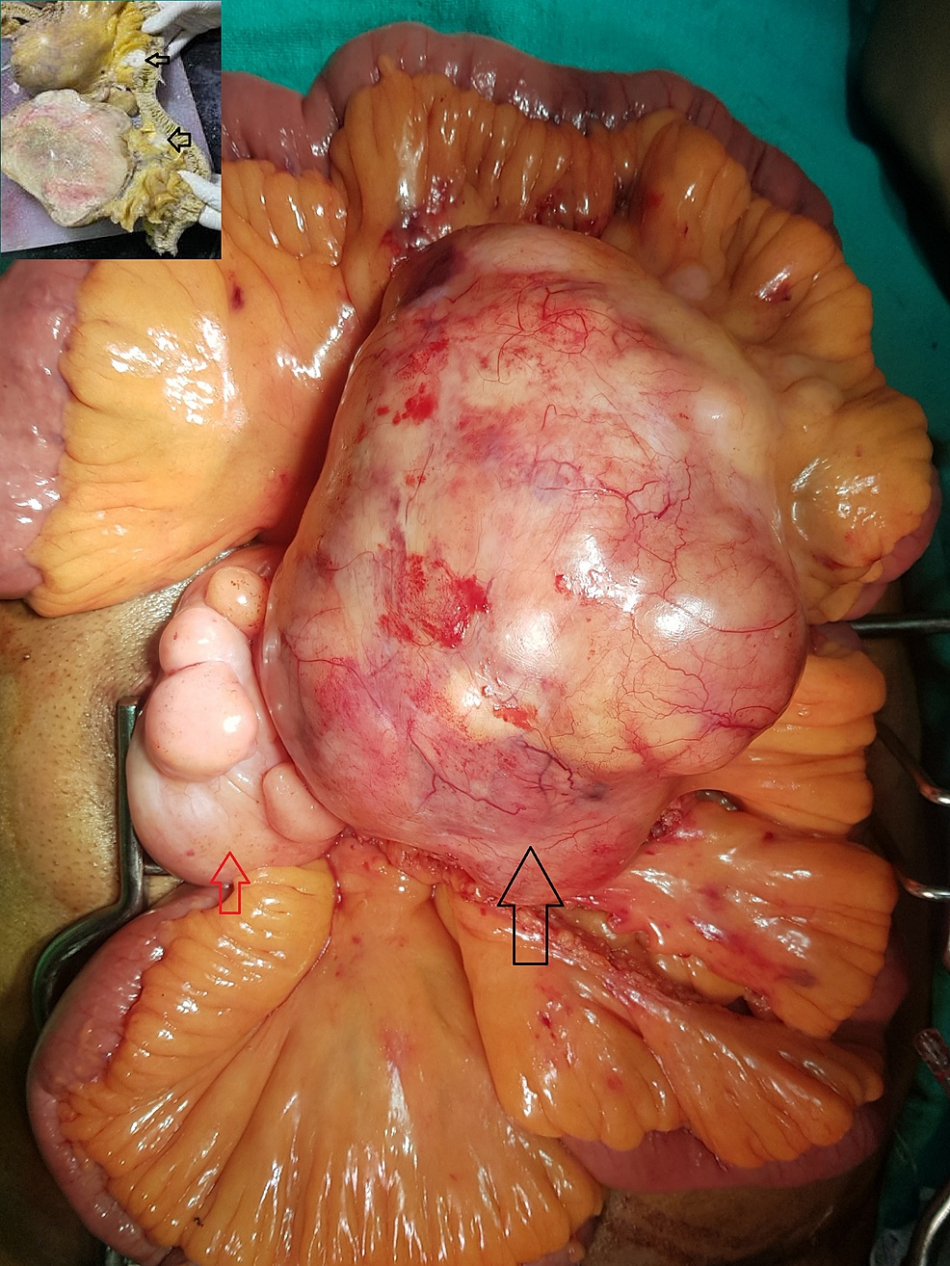
Intraoperative photograph showing the large mass (black arrow) arising from the jejunal mesentery with adjoining lipomatous lesions (red arrow). The cut section of the resected specimen revealed the presence of separate two small nodules abutting the bowel wall (inset).

Intraoperatively, a large mass arising from the jejunal mesentery was identified (Figure 2). Multiple lipomas of different sizes with the largest measuring 6 x 7 x 8 cm were present adjoining the tumor in the jejunal mesentery. Complete en bloc R0 resection of the tumor with the adjacent jejunal loop and end-to-end jejuno-jejunostomy was performed. The operative time was 165 minutes with blood loss of 100 ml. Postoperative course was uneventful with the hospital stay of eight days.

**Figure 3:**
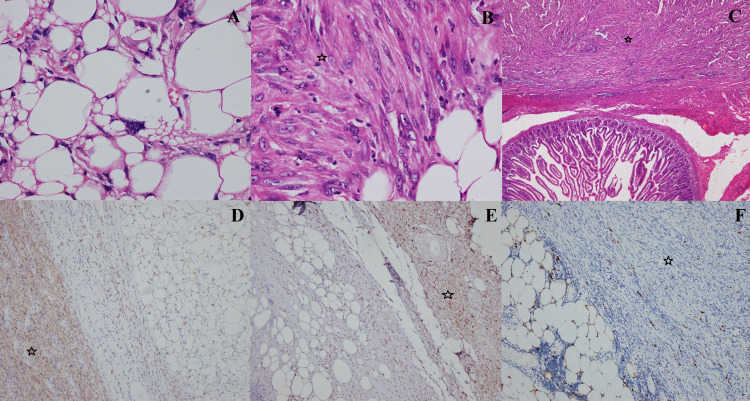
Microscopic examination of the mesenteric mass showing variably sized lipoblasts with vacuolated cytoplasm, central hyperchromatic nuclei and nuclear scalloping (A). The dedifferentiated sarcomatous areas (marked with stars) showed spindle cells with moderate pleomorphism, atypia and brisk mitosis (B). The separate nodules abutting the bowel wall showed spindle cell sarcomatous areas (C). On immunohistochemistry, the tumor cells showed positive staining with CDK4 (D), MDM2 (E) and negative staining with S-100 (F). CDK4 – cyclin-dependent kinase 4, MDM2 – mouse double minute 2.

On the cut section, the large mass had lobulated variegated appearance with small areas of hemorrhage (Figure 2 inset). Microscopic examination revealed features of liposarcoma with variably sized lipoblasts having uni- or multi-vacuolated cytoplasm, central hyperchromatic nuclei and nuclear scalloping (Figure 3A). There was an abrupt transition from the areas of liposarcoma to dedifferentiated high-grade sarcomatous areas. The dedifferentiated sarcomatous areas showed spindle cells with moderate pleomorphism, atypia and brisk mitosis (Figure 3B). The mitotic count was 30-32/10 high-power field. Two small separate nodules were seen abutting the bowel wall (Figure 2 inset). These nodules showed spindle cell sarcomatous areas (Figure 3C). 

The adjoining lipomatous lesions showed mature fat cells with thin capsule. The extent of necrosis was 20%-25%. The histologic grade (French Federation of Cancer Centers Sarcoma Group) was grade 3. The stage of the disease was pT2bNxM0. All the resection margins were free of tumor. The dedifferentiated areas in both the large and small nodules were positive for CDK4 and MDM2 while negative for CD117, DOG1, CD34, SMA, desmin and S100 on the immunohistochemical analysis (Figures 3D-3F). The final diagnosis of multifocal dedifferentiated liposarcoma (DDLS) was made. At the last follow-up two months after surgery, the patient is doing well without any recurrence and receiving adjuvant therapy with ifosfamide and doxorubicin in view of high risk of recurrence. 

## Discussion

DDLS is a rare pathological variant of liposarcoma. It was first described by Evans in 1979 [5]. It consists of a mixture of atypical lipomatous tumor/well-differentiated liposarcoma and high-grade non-lipogenic sarcoma with an abrupt transition between the two components. The dedifferentiated areas often show the histological features of malignant fibrous histiocytoma or low-grade spindle cell sarcoma as seen in the present case. Well-differentiated liposarcoma and DDLS show amplification of several genes including MDM2 and CDK4 [6]. Identification of these gene amplifications by immunohistochemistry, fluorescence in situ hybridization (FISH) or quantitative polymerase chain reaction (PCR) can aid in making the accurate diagnosis as seen in the present case [7].

DDLS typically occurs in the extremities and retroperitoneum similar to the other variants of liposarcoma. Mesenteric liposarcomas are rare. In most of the cases, it presents as a unicentric disease. However, there have been few reported cases of multiple or multifocal mesenteric DDLS as seen in the index case [2,8]. Females are more commonly affected than males [2]. Most of the patients present with abdominal pain and lump as seen in the present case. CECT abdomen and pelvis is the investigation of choice for the preoperative diagnosis of intra-abdominal liposarcoma. In most of the reported cases of mesenteric DDLS, the tumor size has been more than 15 cm due to delayed presentation [2]. The most commonly used staging system for liposarcoma is the AJCC staging system which is based on the tumor size, regional lymphadenopathy and distant metastasis and the most common grading system is the FNCLCC grading system which is based on the degree of tumor differentiation, mitotic count and extent of tumor necrosis.

Complete surgical excision is the mainstay of treatment for mesenteric DDLS similar to that of other types of liposarcoma. Adjuvant chemotherapy for soft tissue sarcoma in adults has been shown to cause a significant improvement in the overall recurrence-free survival [9]. However, studies on mesenteric liposarcoma are lacking due to its rarity. DDLS has a protracted clinical course with approximately 40% chance of local recurrence, 17% chance of metastasis and 28% chance of disease-specific mortality [1,10]. However, there have been anecdotal reports of five-year recurrence-free survival after surgery without adjuvant therapy [3].

## Conclusions

The mesentery is a rare site of DDLS. DDLS should be included in the differential diagnosis of patients with mesenteric lipomatous tumor. Mesenteric DDLS can be multifocal. Complete surgical excision is the only effective treatment of DDLS. Appropriate preoperative surgical planning should be performed to achieve R0 resection. The histopathological examination of the resected specimen is essential to make the definitive diagnosis. Adjuvant chemotherapy may be considered for mesenteric DDLS with a high risk of recurrence.
